# Evaluation of a COVID-19 Vaccine Campaign and SARS-CoV-2 Infection and Mortality Among Adults Aged 60 Years And Older in a Middle-Income Country

**DOI:** 10.1001/jamanetworkopen.2021.30800

**Published:** 2021-10-29

**Authors:** Alejandro Macchia, Daniel Ferrante, Patricia Angeleri, Cristián Biscayart, Javier Mariani, Santiago Esteban, Manuel Rodríguez Tablado, Fernán González Bernaldo de Quirós

**Affiliations:** 1 Subsecretaría de Planificación Sanitaria, Ministerio de Salud de la Ciudad de Buenos Aires, Ciudad Autónoma de Buenos Aires, Argentina; 2 Dirección General de Docencia, Investigación y Desarrollo Profesional, Ministerio de Salud de la Ciudad de Buenos Aires, Ciudad Autónoma de Buenos Aires, Argentina; 3 Gerencia Operativa de Gestión de Información y Estadísticas de Salud Dirección General de Sistemas de Información Sanitaria Subsecretaría de Planificación Sanitaria Ministerio de Salud, Ciudad Autónoma de Buenos Aires, Argentina; 4 Minister of Public Health of the Autonomous City of Buenos Aires, Argentina

## Abstract

**Importance:**

Although there are reports of COVID-19 vaccine implementation in real-world populations, these come from high-income countries or from experience with messenger RNA technology vaccines. Data on outcomes of vaccine deployment in low- or middle-income countries are lacking.

**Objective:**

To assess whether the pragmatic application of the 3 COVID-19 vaccines available in Argentina, 2 of which have no reports of evaluation in real-world settings to date, were associated with a reduction in morbidity, all-cause mortality, and mortality due to COVID-19.

**Design, Setting, and Participants:**

This cohort study used individual and ecological data to explore outcomes following vaccination with rAd26-rAd5, ChAdOx1, and BBIBP-CorV. To correct for differences in exposure times, results are shown using incidence density per 100 000 person-days from the start of the vaccination campaign (December 29, 2020) to the occurrence of an event or the end of follow-up (May 15, 2021). Participants included 663 602 people aged at least 60 years residing in the city of Buenos Aires, Argentina. Statistical analysis was performed from June 1 to June 15, 2021.

**Main Outcomes and Measures:**

Diagnosis of COVID-19 confirmed by reverse transcription–polymerase chain reaction, death from all causes, and death within 30 days of a diagnosis of COVID-19. Poisson regression models were fitted to estimate associations with all 3 outcomes.

**Results:**

Among 663 602 residents of the city of Buenos Aires included in the study, 540 792 (81.4%) were vaccinated with at least 1 dose, with 457 066 receiving 1 dose (mean [SD] age, 74.5 (8.9) years; 61.5% were female [n = 281 284]; 68.0% [n = 310 987] received the rAd26-rAd5 vaccine; 29.5% [n = 135 036] received ChAdOx1; 2.4% [n = 11 043] received BBIBP-CorV) and 83 726 receiving 2 doses (mean [SD] age, 73.4 [6.8] years; 63.5% were female [n = 53 204]). The incidence density of confirmed COVID-19 was 36.25 cases/100 000 person-days (95% CI, 35.80-36.70 cases/100 000 person-days) among those who did not receive a vaccine, 19.13 cases/100 000 person-days (95% CI, 18.63-19.62 cases/100 000 person-days) among those who received 1 dose, and 4.33 cases/100 000 person-days (95% CI, 3.85-4.81 cases/100 000 person-days) among those who received 2 doses. All-cause mortality was 11.74 cases/100 000 person-days (95% CI, 11.51-11.96 cases/100 000 person-days), 4.01 cases/100 000 person-days (95% CI, 3.78-4.24 cases/100 000 person-days) and 0.40 cases/100 000 person-days (95% CI, 0.26-0.55 cases/100 000 person-days). COVID-19–related-death rate was 2.31 cases/100 000 person-days (95% CI, 2.19-2.42 cases/100 000 person-days), 0.59 cases/100 000 person-days (95% CI, 0.50-0.67 cases/100 000 person-days), and 0.04 cases/100 000 person-days (95% CI, 0.0-0.09 cases/100 000 person-days) among the same groups. A 2-dose vaccination schedule was associated with an 88.1% (95% CI, 86.8%-89.2%) reduction in documented infection, 96.6% (95% CI, 95.3%-97.5%) reduction in all-cause death, and 98.3% (95% CI, 95.3%-99.4%) reduction in COVID-19–related death. A single dose was associated with a 47.2% (95% CI, 44.2%-50.1%) reduction in documented infection, 65.8% (95% CI, 61.7%-69.5%) reduction in all-cause death, and 74.5% (95% CI, 66%-80.8%) reduction in COVID-19–related death.

**Conclusions and Relevance:**

This study found that within the first 5 months after the start of the vaccination campaign, vaccination was associated with a significant reduction in COVID-19 infection as well as a reduction in mortality.

## Key Points

**Question:**

Is pragmatic, large-scale use of rAd26-rAd5, ChAdOx1, and BBIBP-CorV COVID-19 vaccines associated with reduction of morbidity, all-cause mortality, and COVID-19–related mortality in a population of individuals aged at least 60 years?

**Findings:**

In this cohort study of 663 602 participants, the use of COVID-19 vaccines was associated with a significant reduction in all-cause death, COVID-related death, and documented infection with the use of 1 dose and even more with the use of 2 doses.

**Meaning:**

These findings suggest that pragmatic use of available COVID-19 vaccines may significantly reduce morbidity and mortality.

## Teaser

This cohort study examines the association of a COVID-19 vaccine campaign and SARS-CoV-2 infection and mortality among adults aged 60 years and older in the city of Buenos Aires, Argentina.

## Introduction

Since the emergence of clinical trial results, mass vaccination campaigns for the prevention of new SARS-CoV-2 infections have begun in the countries that can count on sufficient vaccine doses. Pragmatically assessing the effectiveness and feasibility of vaccination campaigns in practice is an essential tool as randomized clinical trials strictly select the population to be included and have control over logistics that may not represent what is ultimately implemented in health care practice. Some evaluations of the implementation of a vaccination campaign have been reported.^1,2,3,4^ However, these experiences are limited to high-income countries or vaccines with messenger RNA (mRNA) technology.

The city of Buenos Aires, Argentina, started the vaccination campaign on December 29, 2020, using a staggered priority scheme. The population aged at least 80 years and health personnel were targeted first. At the time of the analysis 3 vaccines were available in Argentina. The vaccines used during the period under review were rAd26-rAd5 (Sputnik V [Gamaleya Institute, Russia]), ChAdOx1 (AstraZeneca/Oxford [UK]), and BBIBP-CorV (Sinopharm/Beijing Institute of Biological Products, China). All required 2 doses separated by an interval of at least 3 weeks. All 3 vaccines have published efficacy and safety reports in major international journals.^5,6,7^ Argentina faced at the time of this report a shortage of sufficient vaccines to fully vaccinate its entire population. For this reason, the national health authorities decided to postpone the application of second doses in order to cover as many people as possible with at least 1 application. The health outcomes of this implementation were also analyzed in this study.

Considering that most of the world's people do not have access to mRNA vaccines and in light of the lack of pragmatic evaluation of their effectiveness on collective health indicators, the present analysis attempts to determine whether the application of the 3 vaccines available in Argentina, 2 of which have no reports, to our knowledge, of evaluation in real-world settings, were associated with a reduction in morbidity, all-cause mortality, and mortality due to COVID-19.

## Methods

This cohort study was outside the national requirement of ethical review and provision of informed consent according to the legal regulations in Argentina. This study followed the Strengthening the Reporting of Observational Studies in Epidemiology (STROBE) reporting guideline.

### Study Design and Population

Using administrative data linkage, a retrospective observational cohort covering all residents aged at least 60 years and in the city of Buenos Aires (663 062 out of 3.08 million inhabitants) was recreated. The record of all vaccinated persons in the city was documented and stored by the city government, which was responsible for the vaccination campaign. All cases of reverse transcription–polymerase chain reaction (RT-PCR)–confirmed COVID-19 as well as all hospitalizations and deaths were registered by the Argentine Integrated Health Information System (SISA) which monitors in real time the evolution of the pandemic.

Two data repositories of the Ministry of Health were used for the analysis. The first was the one that records all the people vaccinated in the city of Buenos Aires. This repository contains demographic information such as age and gender, address, occupation, type and lot of vaccine administered, and date of administration of the doses. The second source of data was SISA for persons residing in the city of Buenos Aires. This data set contains the data of all persons who underwent an RT-PCR test in public or private centers in the city of Buenos Aires, together with the results and hospitalization data and vital information for each of the persons tested. In Buenos Aires, the criteria for the diagnosis of COVID-19 was based on the determination of an RT-PCR, so all cases with a suspected clinical syndrome were evaluated using RT-PCR and the results were reported within 24 hours of the test. In addition, other data sources were used to complement the analysis. In order to complement and double-check the number of people who died during the follow-up, all the updated tables with the records of deaths of residents of the city of Buenos Aires during the period analyzed were made available. Race and ethnicity data were not collected as this was a study that made use of data collected in health care and administrative practice, and these characteristics are not systematically collected.

#### Vaccines Used

The 3 vaccines used in this study were (1) the rAd26-rAd5 vaccine consisting of 2 adenovirus vectors (recombinant Ad26 [rAd26] and Ad5 [rAd5]), both containing the gene coding for the SARS-CoV-2 glycoprotein; (2) the ChAdOx1 vaccine, which uses as a vector a modified adenovirus containing the full-length codon-optimized coding sequence of the SARS-CoV-2 spike protein along with a tissue plasminogen activator (tPA) leader sequence; and (3) the BBIBP-CorV vaccine, which is a monovalent vaccine composed of inactivated SARS-CoV-2 virus antigens. All of these vaccines used in the study required 2 doses separated by an interval of at least 3 weeks.^5,6,7^

### Outcomes

The primary objective of the analysis was to determine the incidence density (expressed in cases/100 000 person-days) of documented COVID-19 infection, all-cause mortality, and deaths within 30 days of a documented COVID-19 diagnosis (used as a proxy for COVID-19–related death) among persons who do not receive a COVID-19 vaccine and those vaccinated with 1 or 2 doses of a COVID-19 vaccine. For this purpose, all persons who were vaccinated were followed from the date of vaccination until the occurrence of any of the aforementioned events. For persons who were vaccinated who had no records of documented infection or hospitalization and who were not in the updated death tables, they were assumed to be alive and the date of data censoring was May 15, 2021. On the other hand, persons who were vaccinated who tested positive for any of the index events were censored as of the date of the event. For the death analysis, all persons were followed up to the latest available date of vital status.

For the calculation of rates among persons who were not vaccinated, an ecological approach was used. For this purpose, the number of persons vaccinated on each of the days considered in this analysis was subtracted from the population denominator and rates were calculated by age group. Since the rates among persons who received the vaccine were recorded individually and nominally, the calculation of the rates for the unvaccinated resulted from all documented cases that were not within the group of vaccinated persons by age group. In addition, an exploratory study was conducted on the relative association with outcomes of each of the vaccines used.

### Statistical Analysis

To avoid dissimilar exposure and follow-up times in the cohort of persons who received the vaccine and those who did not receive the vaccine, the results were analyzed in an incidence density aggregate that expressed the rate per 100 000 person-days of follow-up. For unvaccinated persons, follow-up started from the beginning of the vaccination campaign in the city (December 29, 2020) and extended until the insurgence of a documented diagnosis of COVID-19 or death from any cause or both. For the vaccinated cohort, the follow-up period was similar to the unvaccinated for the period prior to the injection of the first dose. From the application of a first dose and from 15 days after that application, the vaccinated cohort was followed until the insurgence of events or the time of administrative censoring on May 15, 2021.

Rates at the end of each period were compared among those who had 2 doses (defined as those who had 2 actual injections and at least 15 days since the second dose), those who had 1 dose, and those who were unvaccinated using Poisson regression stratified by age group (60-69 years, 70-79 years, and 80 years and older) and gender. The vaccine used age as a continuous variable; gender, and history of COVID were used as covariates in all analyses of vaccinated persons.

For the exploratory analysis of the outcomes associated with each of the vaccines, a Cox regression analysis was performed taking the individual data of each person who was vaccinated. The model used age, gender, epidemiological week, and type of vaccine as covariates. All statistical tests were 2-tailed with *P* < .05 considered statistically significant. R statistical software version 3.6.1 (R Project for Statistical Computing) was used to carry out all statistical analyses from June 1 to June 15, 2021.

## Results

Among 663 602 residents aged at least 60 years in the city of Buenos Aires included in the study, 540 792 people (81.4%) were vaccinated with at least 1 dose from December 29, 2020, to May 15, 2021. The mean (SD) age of the entire cohort was 74.4 (8.7) years. Of those vaccinated, 334 488 were female (61.8%). Those who received 1 dose (n = 457 066) had a mean (SD) age of 74.5 (8.9) years, and 281 284 (61.5%) were female. Those who received 2 doses (n = 83 726) had a mean (SD) age of 73.4 (6.8) years and 53 204 (63.5%) were female; 63.5% (n = 343 226) of the population received rAd26 and rAd5, 25% (n = 135 087) received ChAdOx1, and 11.6% (n = 62 479) received BBIBP-CorV. Among the single-dose population, 29.5% (n = 135 036) received ChAdOx1, 2.4% (n = 11 043) received BBIBP-CorV, and 68.0% (n = 310 987) received rAd26-rAd5. Among those who received 2 doses, 0.1% (n = 51) received ChAdOx1, 61.4% (n = 51 436) received BBIBP-CorV, and 38.5% (n = 32 239) received rAd26-rAd5. The documented rate of previous SARS-CoV-2 infection history was 3.7% (20 298 people) in those who received 1 dose and 3.8% (3223 people) in those who received 2 doses.

A total of 148 787 people aged 80 years and older lived in the city of Buenos Aires. Among these, 134 249 (90.2%) received 1 dose and 9089 (6.1%) received 2 doses. Among 221 903 people aged 70 to 79 years, 153 207 (69.0%) received 1 dose and 54 441 (24.5%) received 2 doses. Finally, among a total of 292 327 persons aged 60 to 69 years, 169 610 (58.0%) received 1 dose and 20 196 (6.9%) received both doses. There was a documented history of COVID-19 in 4.3% (n = 23 251) of the people. The Table shows the results for the unvaccinated population as well as for those vaccinated with 1 or 2 doses for the 3 outcomes of interest for the entire study population residing in the city of Buenos Aires.

**Table.** Incidence Density of Confirmed COVID-19 Diagnosis, All-Cause Death, and Death Within 30 Days of a COVID Diagnosis

Age category, y	Outcome	Not vaccinated			One dose			Two doses		
		Cases	Persons/d	Incidence density (95% CI)	Cases	Persons/d	Incidence density (95% CI)	Cases	Persons/d	Incidence density (95% CI)
All ≥60	RT-PCR	24 438	67 415 581	36.25 (35.80-36.70)	5720	29 907 372	19.13 (18.63-19.62)	313	7 225 409	4.33 (3.85-4.81)
	All-cause	10 659	90 828 835	11.74 (11.51-11.96)	1199	29 911 893	4.01 (3.78-4.24)	29	7 225 693	0.40 (0.26-0.55)
	Death due to COVID	1557	67 438 462	2.31 (2.19-2.42)	175	29 912 917	0.59 (0.50-0.67)	3	7 225 719	0.04 (0.00-0.09)
≥80	RT-PCR	2656	10 946 301	24.26 (23.34-25.19)	1956	9 524 737	20.54 (19.63-21.45)	28	996 922	2.81 (1.77-3.85)
	All-cause	4557	20 379 262	22.36 (21.71-23.01)	847	9 525 846	8.89 (8.29-9.49)	13	996 937	1.30 (0.60-2.01)
	Death due to COVID	648	10 948 309	5.92 (5.46-6.37)	138	9 526 555	1.45 (1.21-1.69)	2	996 948	0.20 (0.00-0.48)
70-79	RT-PCR	6256	21 735 880	28.78 (28.07-29.50	2540	14 771 244	17.20 (16.53-17.86)	112	6 440 120	1.74 (1.42-2.06)
	All-cause	3404	30 397 307	11.20 (10.82-11.58)	297	14 773 487	2.01 (1.78-2.24)	13	6 440 219	0.20 (0.09-0.31)
	Death due to COVID	592	21 741 544	2.72 (2.50-2.94)	37	14 773 747	0.25 (0.17-0.33)	0	6 440 232	0.00 (0.00-0.00)
60-69	RT-PCR	15 526	34 733 400	44.70 (44.00-45.40)	1224	19 254 214	6.36 (6.00-6.71	173	1 797 962	9.62 (8.19-11.06)
	All-cause	2698	40 052 266	6.74 (6.48-6.99)	55	19 255 383	0.29 (0.21-0.36	3	1 798 132	0.17 (0.00-0.36)
	Death due to COVID	317	34 748 609	0.91 (0.81-1.01)	0	19 255 438	0.00 (0.00-0.00	1	1 798 134	0.06 (0.00-0.17)

Abbreviation: RT-PCR, reverse transcription–polymerase chain reaction.

### Incidence of Confirmed COVID-19

The rate of confirmed COVID-19 was 36.25 cases/100 000 person-days (95% CI, 35.80-36.70 cases/100 000 person-days) among the unvaccinated, 19.13 cases/100 000 person-days (95% CI, 18.63-19.62 cases/100 000 person-days) in those who received 1 dose and 4.33 cases/100 000 person-days (95% CI, 3.85-4.81 cases/100 000 person-days) among those who had completed the vaccination schedule (2 doses). This was associated with an 88.1% (95% CI, 86.8%-89.2%) decrease in infection rate for those who received the full vaccination schedule (2 doses) and 47.2% (95% CI, 44.2%-50.1%) reduction for those who received 1 dose (Table and Figure).

**Figure.**
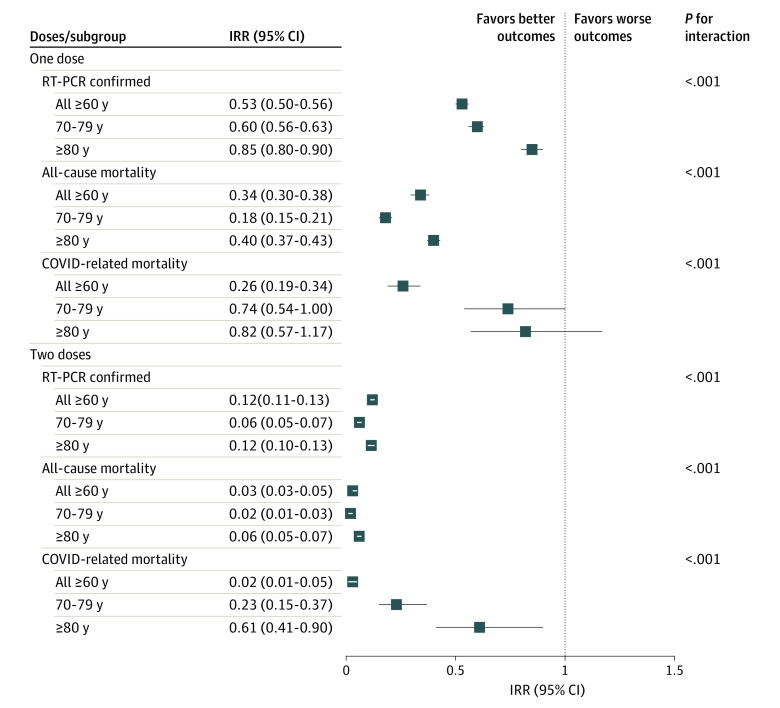
Association of 1 and 2 Doses on Documented Infection, All-Cause Death, and Deaths Temporally Related to COVID-19 by Age Group IRR indicates incidence rate ratio.

For the specific groups of persons aged 70 to 79 years, the full schedule was associated with 94% (95% CI, 93%-94.8%) reduction in infection. For persons aged at least 80 years, it was 88.4% (95% CI, 86.9%-89.8%) (Figure).

### All-Cause Mortality and COVID-19–Related Mortality

The incidence density of all-cause death was 11.74 cases/100 000 person-days (95% CI, 11.51-11.96 cases/100 000 person-days) among unvaccinated persons, 4.01 cases/100 000 person-days (95% CI, 3.78-4.24 cases/100 000 person-days) among those who received 1 vaccine dose, and 0.40 cases/100 000 person-days (95% CI, 0.26-0.55 cases/100 000 person-days) among those who received 2 doses. A single dose was associated with a 65.8% (95% CI, 61.7%-69.5%) reduction in mortality, and the full vaccination schedule of 2 doses was associated with a 96.6% (95% CI, 95.3%-97.5%) reduction (Table and Figure).

The full vaccination schedule was associated with a reduction in all-cause death in all subgroups with a reduction of 94.2% (95% CI, 93.1%-95.1%) among those aged at least 80 years and a reduction of 98.2% (95% CI, 97.2%-98.9%) among those aged 70 to 79 years (Figure).

The same was observed for death within 30 days of a COVID-19 diagnosis, which was 2.31 cases/100 000 person-days (95% CI, 2.19-2.42 cases/100 000 person-days) among unvaccinated persons, 0.59 cases/100 000 person-days (0.5-0.7 cases/100 000 person-days) among persons who received 1 dose, and 0.04 cases/100 000 person-days (0-0.01 cases/100 000 person-days) among persons who received 2 doses. There was a reduction in COVID-related death of 98.3% (95% CI, 95.3%-99.4%) for the full vaccination schedule (2 doses) and 74.5% (95% CI, 66%-80.8%) for people with 1 dose (Table and Figure).

### Outcomes Associated With Each Vaccine

There were no significant differences in the 3 outcomes of interest between people who received 2 doses of rAd26-rAd5 and ChAdOx1 vaccines. Compared with people who received 2 doses of rAd26-rAd5, people who received 2 doses of ChAdOx1 had a similar risk of documented COVID infection at follow-up (hazard ratio [HR], 1.05; 95% CI, 0.80-1.37; *P* = .74) and of death from any cause (HR, 0.69; 95% CI, 0.33-1.45; *P* = .33). Those who received the full BBIBP-CorV schedule (2 doses) had a significantly higher risk in terms of documented COVID-19 infection (HR, 1.65; 95% CI, 1.40-1.93]; *P* < .001) but no statistically significant difference in all-cause mortality (HR, 2.05; 95% CI, 0.79-5.30; *P* = .14).

## Discussion

In most high-income countries that have secured an overabundant supply of vaccines, demonstration of efficacy in clinical trials was followed by a quick pragmatic assessment of effectiveness in so-called real-world settings.^1,2,3,4^ However, most of the world lacks both a reassuring stock of vaccines and the same products that were evaluated in real-world, large-scale care settings. There is, to our knowledge, no assessment of the pragmatic effectiveness of the vaccines they use in low- and moderate-income countries.

In a context of vaccine shortages for low- and middle-income countries, this study found that pragmatic implementation of a vaccination schedule with a group of vaccines that are not existing options (except for the one produced by the Astra Zeneca consortium and the University of Oxford) in high-income countries were associated with a dramatic reduction in documented COVID-19 infection, as well as in all-cause death and deaths temporally related to a COVID-19 diagnosis. This information is of health relevance and this relevance far exceeds the interest of the city of Buenos Aires. In a context where the debate over which vaccine to use has almost as much relevance as how to effectively reach a critical mass of people with vaccines, our results lend support to recent observations from Brazil.^8^ The experience of the small town of Serrana showed that the rapid and massive use of a vaccination campaign with drugs that have an admittedly lower effectiveness in clinical trials than the vaccines used massively in Europe and the United States suggests that the priority should be access over product.

In the experience of the city of Buenos Aires, the administration of 1 dose was associated with moderate prevention of documented infection but was good enough for the prevention of all-cause mortality and death in the proximity of a COVID-19 diagnosis. Although the protection associated with 1 dose in people aged 80 years and older did not achieve a statistically significant decrease in death from COVID-19, 1 dose was associated with a decrease in death from all causes. This is probably because cause of death attribution is always a less rigorous outcome than all-cause death.^9^ It is possible that older people dying from COVID-19 outside a hospital setting may not have been diagnosed in a timely manner. In fact, it is likely that in the older population the documentation of the diagnosis was underestimated. However, in all groups, a single dose was associated with reduced mortality in line with the finding of a reduction in documented infection.

As expected from the results of the clinical trials, a full schedule was associated with twice the prevention of diagnosed disease and a reduction of death with COVID-19 and death from all causes. It should be noted that the outcome assessed in this study is that of documented infection and not asymptomatic infection. People tested and reported to the national data system overwhelmingly do so because of suspected disease based on symptoms and not on population-based screening.

The results should also be discussed from the perspective of the administration of the shortage. Unfortunately, the number of available vaccines was not yet sufficient to vaccinate the population aged at least 60 years with 2 doses. In fact, until mid-May 2021, only about 1 in 5 persons had 2 doses. Despite this, the reduction of events with 1 dose was satisfactory, which supports the thesis that suggests delaying a second dose to increase the number of people with at least 1 injection.

The analysis found no significant protection associated with 2 doses of any of the vaccines used for the prevention of death from any cause. However, compared with the other 2 vaccines, the BBIBP-CorV vaccine was associated with a higher risk of infection during follow-up. The reasons why 2 doses of BBIBP-CorV were associated with less protection against infection remain speculative. It has been suggested that the low efficacy found in people with inactivated whole virus vaccines should not be surprising, as the chemical or physical treatment used to eliminate infectivity may be sufficiently damaging to modify immunogenicity, especially of the antigens needed to elicit cell-mediated immune responses. For this reason, it has been postulated that up to 3 doses of such vaccines may even be necessary.^10^ Additionally, it should be noted that although there was no statistically significant difference in the estimated mortality among the populations that received the different vaccines in this study, this should be evaluated prospectively with larger numbers of cases and events to verify if there is a different effectiveness among these vaccines.

The present study only focused on the analysis of population groups that had a high rate of vaccination with at least 1 dose. Although the first vaccinated population in Argentina was health care workers aged 20 to 59 years, they represent a small fraction of the population and the unvaccinated groups of people of that age have an exposure and risk profile that is quite different from those vaccinated. These differences should be smaller in the analysis presented here, as in the age group of people aged at least 60 years, people with at least 1 dose represent more than two-thirds of the total population in this age group, reaching more than 90% in those aged at least 80 years.

### Limitations

Although analyses with ecological approximations have inherent risks^11^ owing to the lack of correction models that take into account variables associated with the unvaccinated population that may contribute to the differences between groups, the present analysis considered important information to qualify the associations between vaccination and outcomes. First, the results were restricted to a population group with a high vaccination rate that, by definition, represents the majority of the group. Additionally, the analyses were all corrected for gender and age strata as well as for the presence of COVID-19 prior to vaccination. In any case, the results do not claim efficacy as this can only be demonstrated in properly conducted clinical trials. It should also be noted that the study conducted is underpowered to find associations, especially with fatal events, so the results should be interpreted with caution

The relative performance results of each of the vaccines used should be read with caution. The allocation of vaccines was not random and different age groups were vaccinated with different vaccines at different times. Although the regression models take into account these confounding variables, a residual confounding effect cannot be ruled out.

## Conclusions

The present analysis shows that the pragmatic implementation of a vaccination plan including 3 different vaccine options in older adults in the City of Buenos Aires was associated with a significant reduction in documented COVID-19 infection and death from any cause as well as death presumably associated with COVID-19. These results suggest the need to implement mass vaccination strategies with the vaccines that each country has available in the shortest possible time.
